# Simultaneous X-ray diffraction and phase-contrast imaging for investigating material deformation mechanisms during high-rate loading

**DOI:** 10.1107/S1600577514022747

**Published:** 2015-01-01

**Authors:** M. Hudspeth, T. Sun, N. Parab, Z. Guo, K. Fezzaa, S. Luo, W. Chen

**Affiliations:** a Purdue University, West Lafayette, IN 47907, USA; b Advanced Photon Source, Argonne National Laboratory, Argonne, IL 60439, USA; c The Peac Institute of Multiscale Sciences, Chengdu, Sichuan 610207, People’s Republic of China

**Keywords:** Kolsky bar, dynamic diffraction

## Abstract

Using a high-speed camera and an intensified charge-coupled device (ICCD), a simultaneous X-ray imaging and diffraction technique has been developed for studying dynamic material behaviors during high-rate tensile loading. A Kolsky tension bar has been used to pull samples at 1000 s^−1^ and 5000 s^−1^ strain-rates for super-elastic equiatomic NiTi and 1100-O series aluminium, respectively. By altering the ICCD gating time, temporal resolutions of 100 ps and 3.37 µs have been achieved in capturing the diffraction patterns of interest, thus equating to single-pulse and 22-pulse X-ray exposure. Furthermore, the sample through-thickness deformation process has been simultaneously imaged *via* phase-contrast imaging. It is also shown that adequate signal-to-noise ratios are achieved for the detected white-beam diffraction patterns, thereby allowing sufficient information to perform quantitative data analysis diffraction *via* in-house software (*WBXRD_GUI*). Of current interest is the ability to evaluate crystal *d*-spacing, texture evolution and material phase transitions, all of which will be established from experiments performed at the aforementioned elevated strain-rates.

## Abstract 2

A simultaneous X-ray imaging and diffraction technique has been developed for studying dynamic material behaviors during high-rate tensile loading provided by a miniature Kolsky bar.

## 1. Introduction   

In any high-rate loading environment, for example, penetration, blast loading or impact, material deformation and resulting stress-states cannot be described by simple quasi-static testing parameters. It becomes evidently apparent that dynamic loading processes are inherently complex in nature, as simple rigid-body motion gives way to wave propagation effects. Furthermore, in efforts to properly understand a dynamic loading condition, it is of great importance to ascertain the deformation response of each interacting material in loading regimes similar to those experienced in the event of interest, thereby giving constitutive material properties which can ultimately be used in a modeling scheme. Thus, material property characterization is routinely performed with Kolsky bar analysis or flyer plate impact. The former method loads a material under a uniaxial stress condition thereby providing stress–strain response at strain rates in the 10^2^–10^4^ s^−1^ range, but, for special bar geometries, can provide strain rates as low as 10 s^−1^ and as high as 10^5^ s^−1^, also further depending on sample material response and size (Chen & Song, 2011 ▸; Casem *et al.*, 2011 ▸). The latter technique, pressure-shear plate impact, typically yields shear strain-rates in the 10^5^–10^6^ s^−1^ regime but for specific sample geometries (*e.g.* thin films) has been reported as high as 10^7^ s^−1^ (Clifton & Klopp, 1985 ▸). Indeed, a wide array of strain rates can be achieved with proper understanding and utilization of such techniques, although the experimental results provide solely an average deformation within the sample and give no understanding of localized deformation or damage propagation. Barring transparent materials, only surface tracking of damage mechanisms can be performed with high-speed optical imaging, thereby gaining a slight advantage over signal response, yet still limiting analysis onto the sample surface, leaving the interior deformation mechanisms unknown. Recently, an *in situ* imaging technique has been developed wherein through-thickness damage and deformation can be tracked internally within the sample utilizing hard X-rays (Luo *et al.*, 2012 ▸; Gupta *et al.*, 2012 ▸; Hudspeth *et al.*, 2013 ▸; Chen *et al.*, 2014 ▸). Using phase-contrast imaging (PCI), high levels of contrast can be generated for variations in the refractive index of different materials, and successful through-thickness image sequences have been produced for both a variety of material types and structural interactions. Although this is of extreme interest, there is still a vast amount of information that could be generated from analyzing the X-rays which are diffracted by the samples during the dynamic loading process (Turneaure & Gupta, 2009 ▸; Turneaure *et al.*, 2009 ▸). Historically, this sort of experiment has been performed on samples undergoing stepwise strain states or, at best, very low quasi-static loading conditions. With this sort of loading history, determination of crystal *d*-spacing, texture evolution and phase transitions can be analyzed, but unfortunately, as was stated, is inherently limited to static stress–strain states. Even though such quasi-static measurements provide a baseline understanding of the effect of loading processes on different materials or structural types, it does not allow for a richer understanding of the complex mechanisms presented during a dynamic stress sequence. For example, it has been shown that the phase change occurring during Kolsky bar loading in equiatomic NiTi displays an extreme amount of rate sensitivity (Chen *et al.*, 2001 ▸). The sharp transition front (Young *et al.*, 2010 ▸) which sweeps across the sample becomes either smeared or multiple transition zones develop, which negate the elastic-plastic elastic stress–strain response inherent for this material when tested at quasi-static conditions, thereby yielding a mixed elastic plastic response in the phase transition regime (Chen *et al.*, 2001 ▸; Nemat-Nasser *et al.*, 2005*a* ▸,*b* ▸). With high-speed diffraction, such transient phase change can be probed in real time. Furthermore, as previously stated, one could use the diffracted beam to gain insight into crystalline *d*-spacings and texture evolution of dynamically loaded samples, thereby aiding in the understanding of deformation mechanisms of the elastic regime in rate-sensitive materials. It is therefore the purpose of this work to show the possibility of performing such high-strain-rate diffraction experiments on two different material types, namely aluminium 1100-O and super-elastic equiatomic NiTi when being loaded in a dynamic tensile environment. A miniature tension Kolsky bar has been used to provide loading to these materials in efforts to display the peak position change of certain crystal directions in the Al samples, and the stark phase change from austenite to martensite that occurs in the NiTi material.

## 2. Experimental   

### 2.1. X-ray source   

Kolsky bar experiments are inherently dynamic in nature, with the specimen deformation process generally occurring in the realm of 100–200 µs. Thus, as compared with more rapid loading schemes such as shock wave studies developed *via* gas gun experiments, Kolsky bar loading provides comparatively ample deformation time, thereby yielding experiments which are more easily captured in the current timing scheme (details are described in §2.4). That said, these events are still quite rapid, thereby enforcing the implementation of high-energy high-flux X-rays which can penetrate through the sample thickness to generate phase-contrast images and diffraction patterns with a sufficient signal-to-noise ratio. In order to obtain such bright X-rays, the Advanced Photon Source (APS) at Argonne National Laboratory has been utilized, as it produces X-rays with temporal resolutions and energy characteristics of consequence. In the standard run mode offered at APS, 24 equispaced 100 ps-long electron bunches travel around the storage ring with a period of 153 ns, thereby providing ample framing time for Kolsky bar loading. Furthermore, due to the longevity of the experimental duration, multiple X-ray pulses can be used for detection of each image and diffraction frame *via* increasing the camera exposure time.

**Figure 1:**
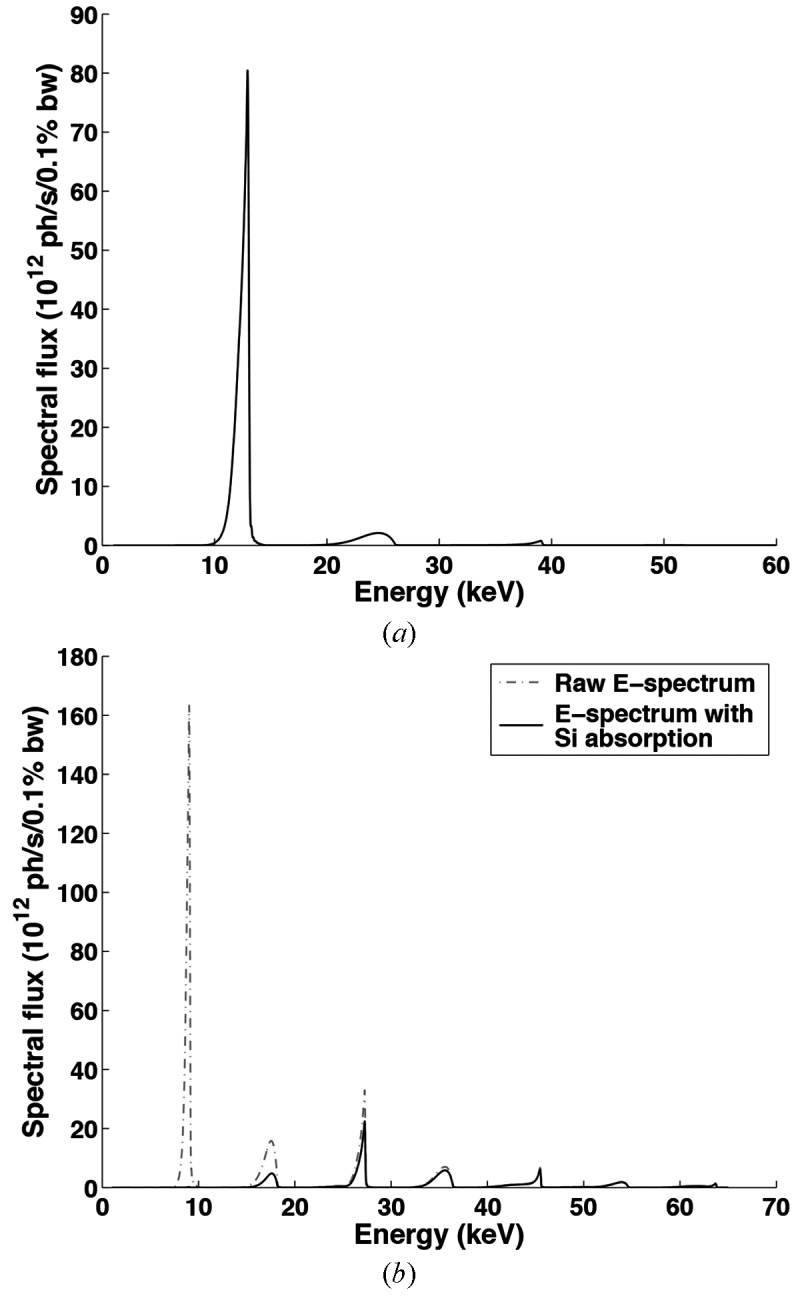
X-ray energy spectrum for the (*a*) aluminium and (*b*) NiTi specimens. The aluminium tests used an undulator gap of 30 mm, and the first harmonics become of most interest. The NiTi samples used an undulator gap of 20 mm, which yields X-rays with much higher flux. In efforts to mitigate this sample absorption effect, a single-crystal Si wafer (1 mm thickness) was placed upstream of the X-ray path to filter out the low-energy photons.

The X-ray beamline utilized for these experiments (32-ID-B) was equipped with APS Undulator A, which possesses a period and length of 3.3 cm and 2.4 m, respectively. A white beam was used so as to increase the X-ray flux for high-speed imaging and diffraction experiments with limited number of pulses. The undulator gap is variable, providing variations in spectral flux along with energy shifts of harmonic peaks. In the present work the undulator gaps of interest were 30 mm and 20 mm, and resulting energy spectrum curves can be found in Fig. 1 ▸. Compared with a monochromatic beam, the flux of a white beam is usually many orders higher, and thereby the beam-induced heating effect needs to be carefully assessed. In a separate experiment with the undulator gap set to 30 mm, we collected single-pulse diffraction patterns from Al samples during *in situ* heating in a helium environment. The results reveal that the measured lattice parameters of the sample at different temperatures match the theoretical values very well. In the temperature range from 298 to 573 K, the largest deviation is only 0.01%, which is much smaller than the magnitude of strain measured in our Kolsky bar experiments. When we reduced the undulator gap to 20 mm in our experiments, a Si single crystal of thickness 1 mm was placed in the upstream beam path to filter out the low-energy photons. As shown in Fig. 1(*b*) ▸, photons with the first-harmonic energies are entirely absorbed by the Si filter. As a result, compared with the beam generated by the undulator with 30 mm gap, the integrated flux of the Si filtered beam is smaller and the harmonic energies are higher. Both of these two factors contribute to an even lower heat load on the sample. Therefore, we believe the beam-induced heating effect in our experiments is negligible.

### 2.2. Imaging and diffraction geometry   

**Figure 2:**
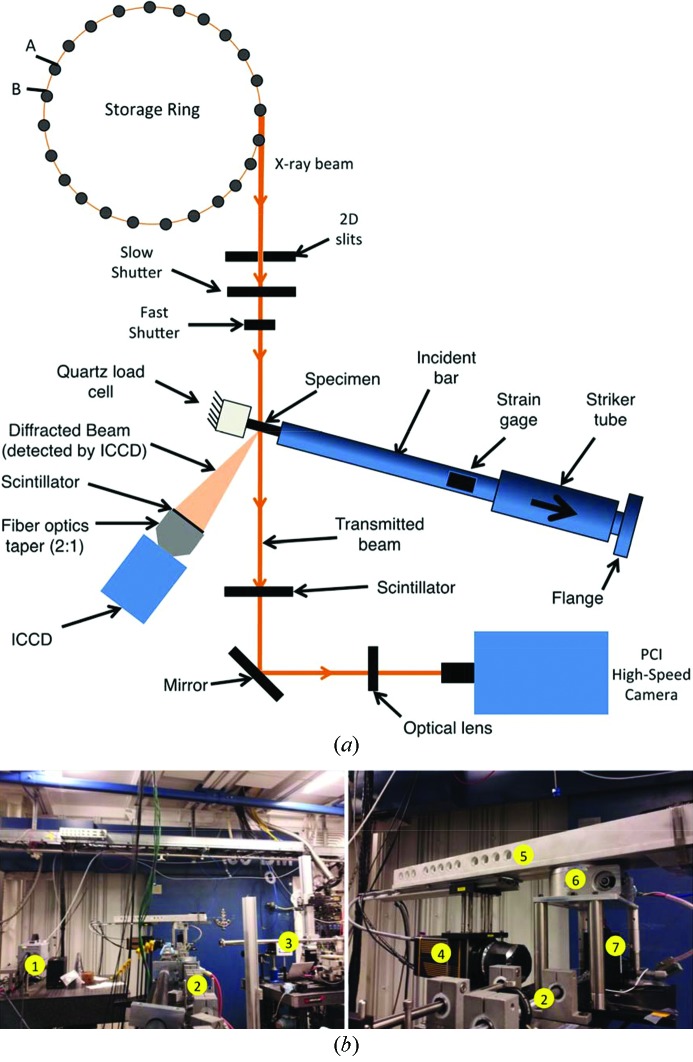
(*a*) Schematic of the integrated X-ray system and Kolsky bar apparatus. Note that the standard 24-bunch mode offered by APS was utilized. In the current configuration, both transmission X-ray diffraction and PCI were detected simultaneously during the high-rate Kolsky bar loading *via* an ICCD and high-speed camera, respectively. (*b*) Photographs of the setup within the 32-ID-B hutch. Demarcations have been placed on vital pieces of equipment in both images including: (1) high-speed camera used for PCI; (2) miniature tension Kolsky bar; (3) helium-filled X-ray flight path; (4) ICCD camera used to record the diffraction pattern during high-rate loading; (5) rotating camera arm; (6) goniometer; and (7) positioning stage used to mount the fast-response load cell. Note that there is a compression Kolsky bar also located on the bar frame, thereby allowing for either tension or compression testing depending on bar selection.

Fig. 2 ▸ shows a schematic and photographs of the experiment setup. In the experiments, both in-line phase-contrast imaging and *in situ* diffraction can be detected simultaneously. The sample orientation and camera geometry have been utilized and prepared so as to allow for transmission diffraction, as the reflection mode requires greater amounts of bar rotation with respect to the beamline, and inherently hinders small-angle detection due to the placement of the backstop and mounted load cell. The phase-contrast images of the samples were collected by an optically coupled high-speed camera (Photron FastCam SA1.1), while the diffraction patterns were recorded using an optically coupled intensified charge-coupled device (ICCD; Princeton Instruments PI-MAX). The scintillators for imaging and diffraction detection are LuAG:Ce (100 µm thickness) and LYSO (300 µm thickness) crystals, respectively. The ICCD was mounted on a rotation arm, which allowed for the camera to be continually located in the typical circular track configuration as compared with an arbitrary position. The circular track configuration could facilitate ease of pre- and post-analysis of white-beam diffraction patterns and control of the detection angle. A Huber 410 goniometer was used to control the rotation arm with an angle resolution of 0.001°. It was possible to adjust the sample-to-detector distance by sliding the camera along a linear rail on the rotation arm; a photograph of the assembly has been included in Fig. 2(*b*) ▸.

**Figure 3:**
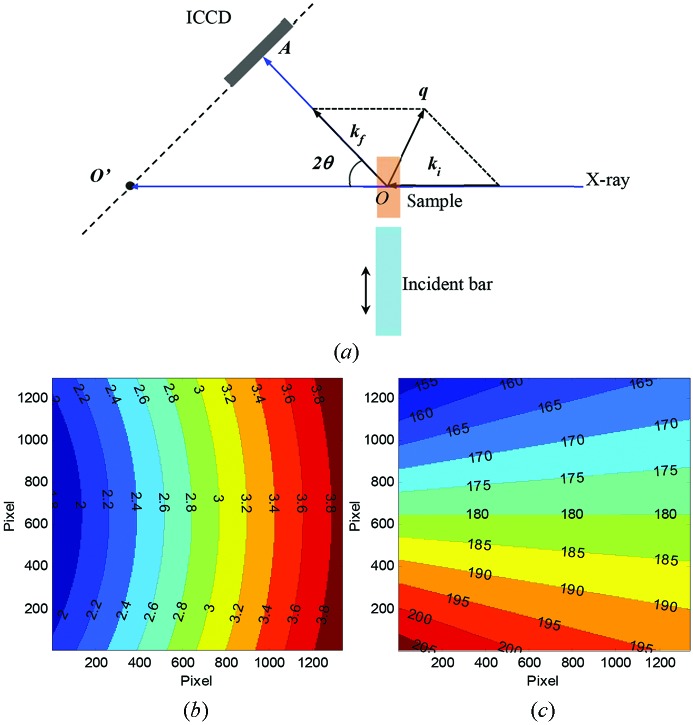
(*a*) Diffraction geometry. *O* is where the X-ray beam and the sample interact; *O*′ is the transmitted beam position on the detector plane; *A* is the scattered beam position on the detector; *k*_i_ and *k*_f_ stand for the wavevectors for the incident and out-going beam, and *q* is the scattering wavevector. 2θ (*i.e.* angle *O′OA*) is the diffraction angle. (*b*, *c*) The pixelated scattering vector *q* map (*b*) and azimuthal angle φ map (*c*) on the ICCD, when the detector rotation angle is 25°, X-ray energy is 12.9 keV and the beam position is assumed to be vertically centered with the detector.

The diffraction work performed in this study was aided *via* the in-house program *WBXRD_GUI*. This Matlab-coded software has been developed for simulating and analyzing white-beam diffraction patterns from polycrystalline samples. It is particularly useful when one deals with (single-pulse) noisy diffraction patterns, and an area detector is placed with an offset angle from the incident direction, *i.e.* the detection plane is not perpendicular to the incident beam while the transmission spot may not fall on the detector, as shown in Fig. 3(*a*) ▸. For a given detector location and X-ray energy, the scattering vector *q* and azimuthal angle φ at each pixel position on the detector can be calculated. Figs. 3(*b*) and 3(*c*) ▸ show examples of *q* and φ maps for a detector angle of 25° and X-ray energy of 12.9 keV (*i.e.* 30 mm undulator gap) assuming the beam position is vertically centered with the detector.

The simulation of a white-beam diffraction pattern from a known material starts from the calculation of monochromatic beam diffraction patterns for the specific detector location, *I*(θ,*E*). Here, the intensity profile is described using the pseudo-Voigt function. The sample crystal structure and the diffraction intensities *I* of different atomic planes (*hkl*) are input parameters, which can be obtained from the International Center for Diffraction Data (*i.e.* JCPDS cards). For each X-ray energy *E*, *WBXRD_GUI* calculates the reciprocal lattices and projects diffraction information onto the detector plane. These mono-beam diffraction patterns are then integrated over the entire energy range with the weighting factor being the flux of photons with different energy, *F*(*E*). Here, *F*(*E*) reflects the post-sample flux density, which has been modified by considering the sample absorption of photons with different energies,
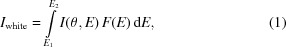
For our case, *E*_1_ and *E*_2_ are typically 1 keV and 60 keV, respectively. Photons with energy higher than 60 keV have much lower flux (Fig. 1 ▸) and the corresponding diffraction scattering angles are very small, thereby their diffraction contribution is not considered. To improve the calculation speed, discrete diffraction peaks are considered, meaning that *I*(θ,*E*) is normally replaced by a series of *I*_*hkl*_(θ,*E*). Equation (1) then becomes
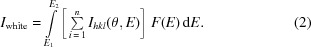
Essentially, the white-beam diffraction intensity at a given scattering angle is the convolution of the input diffraction intensity for different atomic planes with the energy spectrum of the X-rays. Note that the polarization of X-rays is not considered in the simulation.

### 2.3. Kolsky bar apparatus   

In order to provide a dynamic tension loading environment, a modified Kolsky bar was employed. A very basic schematic of the apparatus can be seen in Fig. 2(*a*) ▸ and is thoroughly reviewed elsewhere (Chen & Song, 2011 ▸). The bar material was aluminium and possessed a length and diameter measuring 225 cm and 12.7 mm, respectively. A brass striker tube was used to impact a flange located on the incident bar end, thereby generating an elastic tensile stress pulse. The usual travel time between the solenoid firing signal and striker-bar impact was ∼120 ms and possessed no more than a 10 ms jitter. Typical striking velocities ranged from 3 to 5 m s^−1^. Post-impact, the elastic stress wave was recorded *via* two strain gauges located on the incident bar surface. Location of the strain gauges was specifically designed to ensure that overlap in the incident and reflected pulses would be non-existent and also to ensure a necessary time window elapsed for shutter activation, which is described in §2.4. In this experimental configuration, due to constraints driven by hutch size limitations, the traditional transmission bar was replaced by a Kistler 9712B5 fast-response quartz-based load cell, which is acceptable if the impedance mismatch between the bar end and sample is large (Cheng *et al.*, 2005 ▸; Chen & Song, 2011 ▸). The resulting force transducer output signal was then sensed and amplified by a Kistler 5010 dual mode amplifier. Both the amplified signal generated from the incident bar strain gauges and the transmission load signal were collected using a Tektronix DPO 4032 oscilliscope. Further description of the loading scheme has been described in previous works (Hudspeth *et al.*, 2013 ▸; Chen *et al.*, 2014 ▸).

In order to ascertain the strain rate (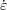) and strain (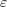) incurred by the dynamically loaded sample, the well known Kolsky bar relations have been utilized and are described in equations (3) and (4),
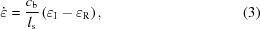


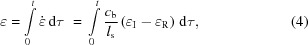
wherein 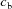, 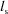, 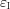, 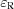 represent the incident bar acoustic wave speed, sample gauge length, incident signal bar strain and reflected signal bar strain, respectively. As previously stated, due to hutch size limitations and the specifically designed large impedance mismatch between the sample and incident bar end, the typical Kolsky transmission bar has been replaced with a fast-response load cell, thereby allowing for direct detection of sample axial force (*F*). This force is divided by the sample initial cross-sectional area (*A*_o_) in order to obtain the average instantaneous longitudinal engineering stress (σ = *F*/*A*_o_) throughout the duration of loading.

**Figure 4:**
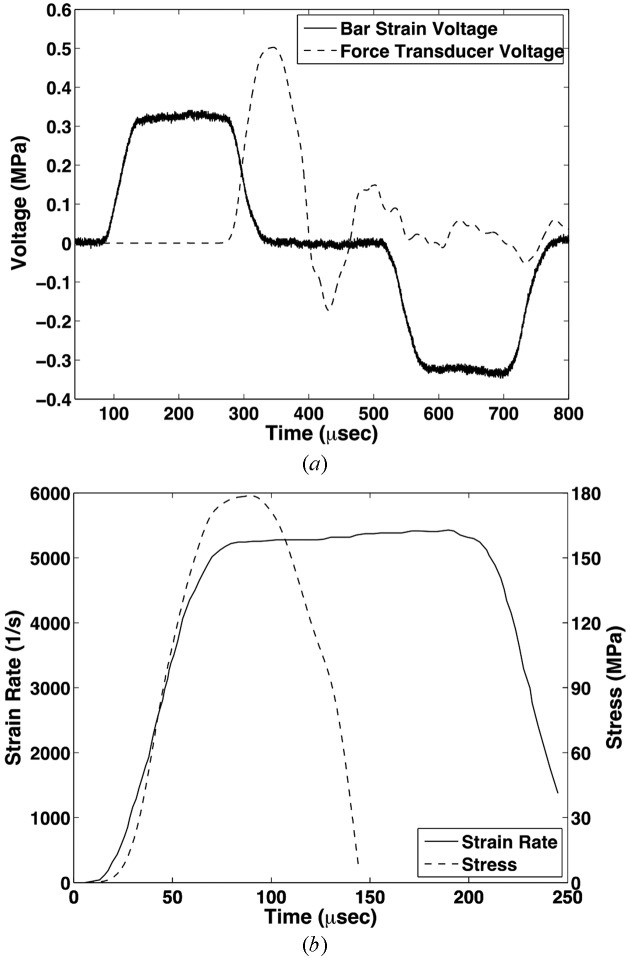
(*a*) Raw voltage signals relevant to the high-rate loading produced from the Kolsky bar setup. Note that *in lieu* of a transmission bar, a fast-response quartz load cell has been utilized, which results in a reduced experimental framework footprint required by the constrained hutch dimensions. This load response is represented by the dotted black line. Furthermore, this load detection approach is viable if the impedance mismatch between the incident bar end and sample is quite large, which is demonstrated by the similarity between the incident and reflected waveforms shown by the solid black line. (*b*) Resulting force and strain-rate histories represented by dotted black and solid black curves, respectively. Note that the sample is loaded into the constant strain-rate regime.

In order to deliver a valid stress–strain response from a loaded material, it is imperative that the strain rate experienced during the experiment be of a constant value. Thus, typical strategic pulse shaping was employed which allows for modification of the incident stress wave shape, ultimately being tailored to meet the response of the tested material with an educated trial-and-error approach (Christensen *et al.*, 1972 ▸; Nemat-Nasser *et al.*, 1991 ▸) or *via* analytical solution (Frew *et al.*, 2002 ▸). In the current experimental design, the cross-sectional area of both the Al and NiTi samples were of such insignificant size with respect to the incident bar that the vast majority of the incident signal is reflected due to the extremely high impedance mismatch. In this case, it is then desired to generate a trapezoidal pulse, and only slight pulse shaping is needed to reduce the high-frequency stress response. A typical set of recorded waveforms can be seen in Fig. 4(*a*) ▸, which demonstrate the trapezoidal shape of the incident and reflected waveforms. Achievement of a constant strain-rate is corroborated by Fig. 4(*b*) ▸, which shows the strain-rate history of the loaded sample along with the specimen stress recorded by the fast-response load cell.

### 2.4. Timing sequence   

**Figure 5:**
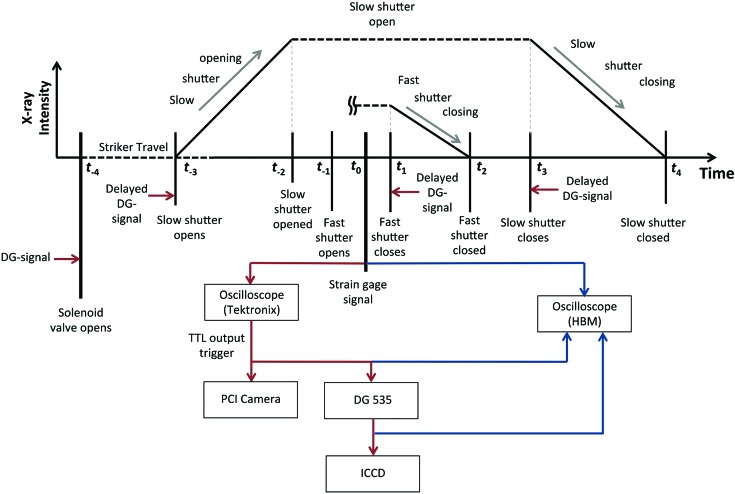
Schematic of the timing sequence used throughout the experimental duration. At time *t*_−4_ a delay generator (DG) signal is sent to open the gas gun valve, thereby firing the striker. Governed by a specified delay dictated from a predetermined striker travel time, an additional DG signal is sent to open the slow shutter at time *t*_−3_, which is the opening bracket for the outer experimental window. Note that the slow shutter opening signal is sent early enough to ensure that the shutter window is completely open before the striker impacts the incident bar end. Upon striker impact, a tensile stress wave is sent down the incident bar and, as this wave passes through the bar at time *t*_0_, it is detected by a set of strain gauges, which are located 84 cm from the sample interface. Upon detection of the stress wave, a delayed DG signal is sent to close the fast shutter system, demarcated by time *t*_1_. Finally, an ultimate DG signal is sent to close the slow shutter system at time *t*_3_, thus closing the outer experimental time window.

In order to protect the scintillator for PCI and reduce unnecessary heat load on the sample, temporal bracketing of the experimental period of interest becomes necessary and is therefore described in detail. Effective timing for experimental delays was achieved *via* careful development and execution of a double-window timing scheme composed of a set of fast shutters and slow shutters. The slow shutters consist of water-cooled bulky copper blocks which can bear the heat load caused by the intense white beam, while the fast shutters are made of small rotating lead blades that open and close much faster than the slow shutters. A schematic of the entire sequence can be seen in Fig. 5 ▸. The external window, referenced from *t*_−3_ until *t*_4_, controls the slow shutter system and is governed by delays referenced from *t*_−4_, namely firing the solenoid, which releases gas into the gun barrel thereby accelerating the striker. This external timing window is designed to possess a slow shutter full opening time of roughly 40 ms (*t*_−2_ to *t*_3_), which can adequately account for the striker to incident bar impact time variation. Inside of the slow shutter open time window lies the internal timing window operating a pair of fast shutters, which further bracket the actual experimental time of interest down to a few milliseconds (*t*_−1_ to *t*_1_). Triggering for the PCI camera was sent immediately at *t*_0_, as the onboard memory storage allowed for ample total time of recording, being much longer than the duration of the entire experimental event. In contrast, triggering of the ICCD camera required special attention as, in the case of this experiment, the system was only able to capture one single frame. A system of sequential delay changes was thus instilled over multiple experimental events so as to build up a diffraction pattern evolution during the entire loading history. As the purpose of this set of experiments was to show the efficacy of achieving a necessary signal-to-noise ratio during small time windows throughout the entire Kolsky bar loading event, this capture method was reasonable, but, upon future work, installation of a multi-frame intensified imaging system would be more fruitful, thereby allowing for the capture of a diffraction pattern evolution during an entire loading sequence. Thus, for each dynamic tensile experiment, a multi-frame PCI sequence was recorded with a high-speed camera, along with a single diffraction pattern that was registered onto an ICCD within the duration of loading. Furthermore, as described in §3.1, single-pulse diffraction was possible for the aluminium samples tested and eight-pulse diffraction was achieved for the NiTi samples, thereby resulting in a possible framing rate of 6.5M frames s^−1^ and 815K frames s^−1^, respectively.

## 3. Results and discussion   

### 3.1. Aluminium   

Inclusion of aluminium 1100-O in this experimental sequence as the first trial material was twofold. First, the material response of Al has been well characterized *via* mechanical loading and thus provides a reasonably well understood stress–strain response at high loading rates (Lindholm & Yeakley, 1968 ▸; Kahn & Huang, 1992 ▸). Second, diffraction analysis of Al embodies a large sect of research, and thus a plethora of diffraction analysis already exists (Kabekkodu, 2010 ▸). Ultimately, the goal of this material class is to show the efficacy of ascertaining strains in specific crystal directions *via* diffraction peak shift.

**Figure 6:**
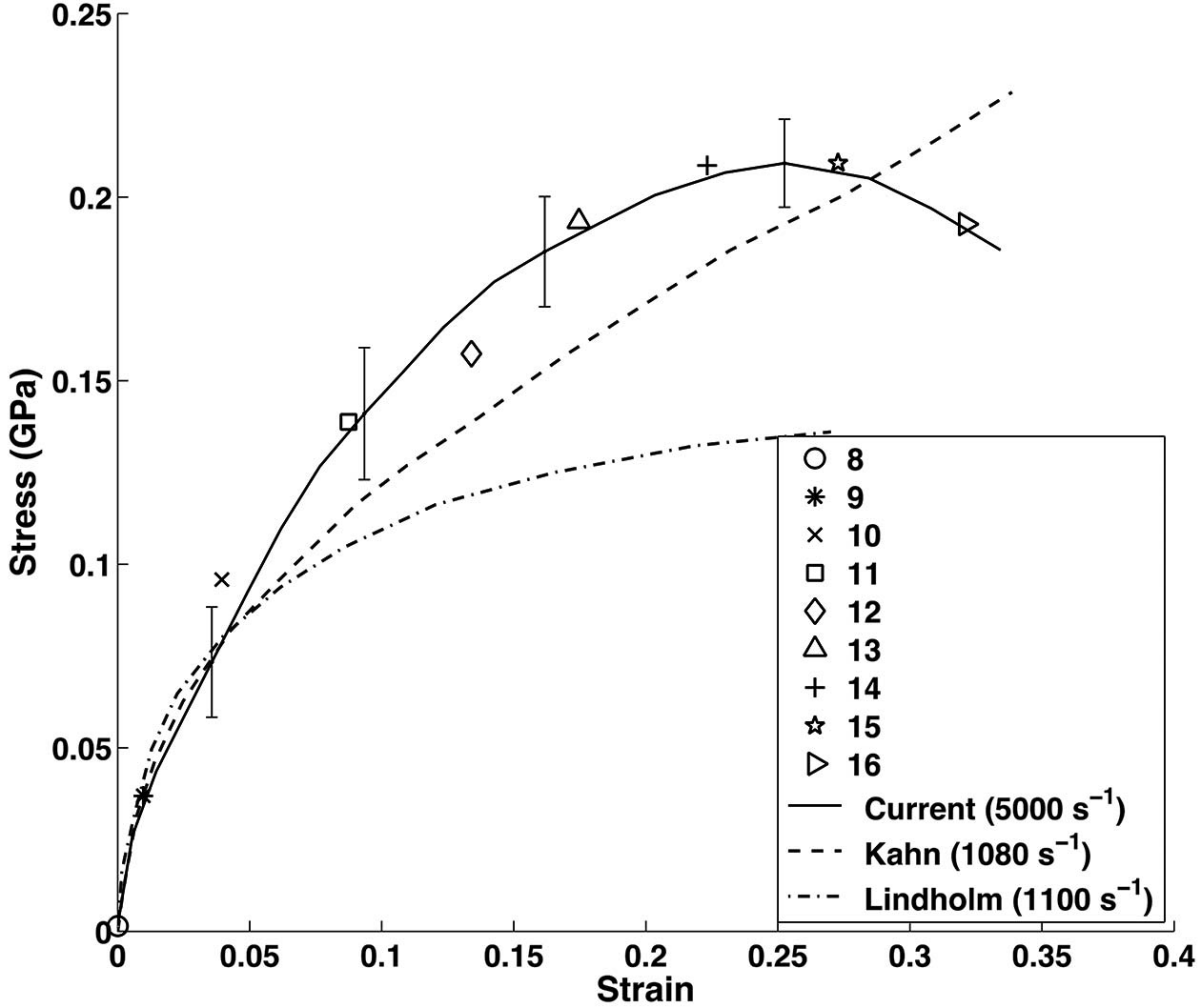
Representative stress–strain curve from the 1100-O aluminium samples pulled in tension. The current data, representing a testing strain-rate of 5000 s^−1^, is demarcated with the solid black curve, and for comparison previous data performed at 1000 s^−1^ (Kahn & Huang, 1992 ▸; Lindholm & Yeakley, 1968 ▸) have also been included. The various symbols which are overlaid on the plot dictate stress–strain states at which a diffraction pattern has been recorded with the ICCD. In-depth analysis from only an unloaded sample and a sample loaded up to demarcation 10 have been included in the in-depth diffraction pattern analysis shown in Fig. 8 ▸, as the current goal of this study is to show the possibility of performing such high-strain-rate loading while simultaneously capturing high-frame-rate diffraction patterns and phase-contrast images.

**Figure 7:**
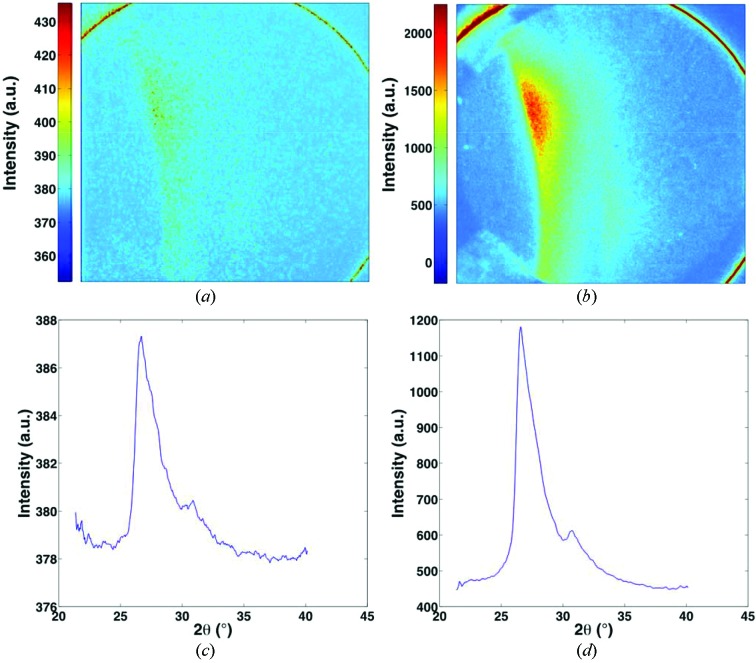
Experimental diffraction patterns wherein the ICCD gating time was varied so as to allow for (*a*) single-pulse diffraction and (*b*) 65-pulse diffraction. Azimuthal integration of each pattern was performed to display the corresponding one-dimensional intensity plots for (*c*) single-pulse diffraction and (*d*) 65-pulse diffraction. Clearly the multi-pulse gating yields a much higher signal-to-noise ratio, but note that there is still enough information in the single-pulse image to detect a distinct ring pattern and well defined peak position *via* post-processing.

In order to generate a high-rate tension loading environment, the miniature Kolsky bar apparatus described in §2.3 was utilized, thereby generating a strain rate in the regime of 5000 s^−1^. An average resulting stress–strain curve is plotted in Fig. 6 ▸, showing agreement with previous work (Lindholm & Yeakley, 1968 ▸; Kahn & Huang, 1992 ▸), wherein the flow stress is higher for the current data due to the rate sensitivity of the material. On the said stress–strain figure, symbols have been plotted which demarcate the strain at which a diffraction pattern was taken. In the current setup, only one pattern was capable of being recorded during the short duration of interest, being 3.37 µs duration for the Al tests, relating to an integration of 22 X-ray pulses. This duration has been chosen to maximize signal intensity, thereby increasing the signal-to-noise ratio and thus allowing for valid diffraction pattern analysis. It is important to note that capturing a single-pulse diffraction pattern was possible for the Al samples, and a resulting pattern was included in Fig. 7(*a*) ▸. For a reference, an Al diffraction pattern generated with 65 X-ray pulses is shown in Fig. 7(*b*) ▸. Note that the intense ring-shape feature appearing at the corners of each pattern is the edge of the scintillator. Both patterns were obtained from 225 µm-thick samples. Furthermore, integration of both patterns was performed to display the corresponding one-dimensional intensity plots and the results are displayed in Figs. 7(*c*) and 7(*d*) ▸, respectively. Pulse duration parameters have also been succinctly included in Table 1 ▸.

**Figure 8:**
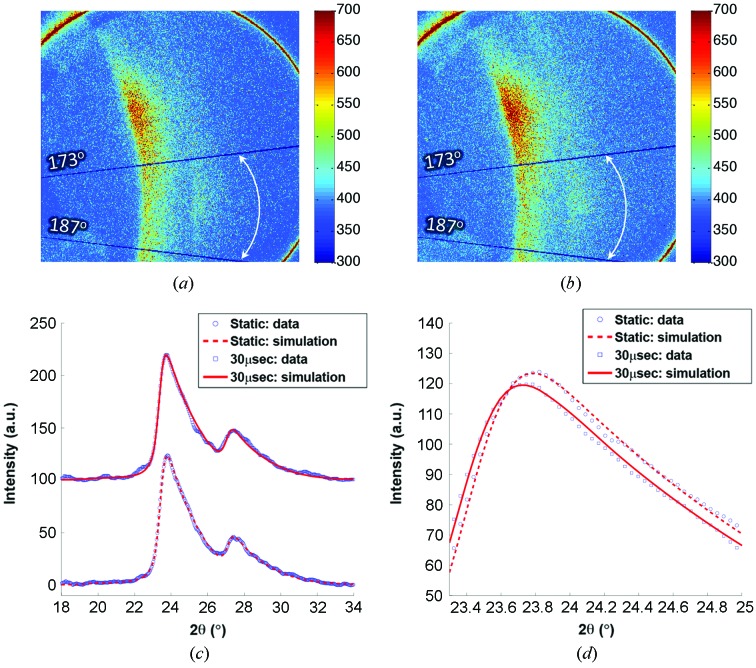
Diffraction patterns from the Al sample, collected (*a*) before and (*b*) 30 µs after the start of tensile pulling. (*c*) Diffraction intensity and corresponding simulations from unstrained and strained Al samples. The one-dimensional data profiles were obtained by radially averaging the two-dimensional diffraction patterns shown in (*a*, *b*) from azimuthal angle 173° to 187°, as indicated. (*d*) A closer look at the (111) peaks, showing the shift of the peak due to a lattice strain of 0.25%. Note the disparity in elastic strain of the (111) peak as compared with the large amount of average plastic tensile strain demonstrated in Fig. 6 ▸.

Figs. 8(*a*) and 8(*b*) ▸ show the diffraction patterns from an Al sample collected before and 30 µs after the start of the tensile pulling, respectively. These patterns were formed by 22 pulses of X-rays (*i.e.* exposure time 3.37 µs) with the energy spectrum shown in Fig. 1(*a*) ▸. In both patterns, two diffraction peaks can be observed. The peak at the lower angle (left) is attributed to Al (111), and the peak at the higher angle (right) is Al (200). Both are generated by X-rays with the first-harmonic energy (12.94 keV). The second-harmonic (311) and (222) peaks are present between these two major peaks. However, as the flux of X-rays with the second-harmonic energy is less than 3% of that of the first-harmonic X-rays, the (311) and (222) peaks are overwhelmed by the intense first-harmonic (111) peak. Fig. 8(*c*) ▸ depicts the one-dimensional diffraction intensity profiles (open symbols) and corresponding theoretical simulations (lines) of unstrained and strained states of the Al sample. The one-dimensional data were obtained by integrating the two-dimensional patterns over the azimuthal angles ranging from 173° to 187°, as indicated in Figs. 8(*a*) and 8(*b*) ▸. As shown in the diffraction geometry (Fig. 3 ▸), diffraction peaks around 0° and 180° azimuthal angles (*i.e.* along the horizontal *q* axis) contain strain information under the present uniaxial stress condition. In the simulations, an anisotropy parameter (*i.e.* I111/I200) was considered to account for the initial texture structure of the Al sample. Fig. 8(*d*) ▸ shows a closer look of the (111) peaks, and a shift of the peak position to the lower angle caused by the tensile stress can be clearly observed. The simulation indicates a 0.25% lattice expansion of the Al sample along the tensile loading direction. The quantitative agreement between the data and simulation demonstrates that ultrafast white-beam diffraction is capable of measuring elastic strain with extremely small magnitude. Thus, rather than solely determining an average strain measure over the entire sample during Kolsky bar loading, it is now possible to additionally analyze elastic strain in a specific loading direction at extremely high temporal resolutions.

### 3.2. NiTi   

Inclusion of the NiTi super-elastic material class was chosen due to the stark phase-change that occurs during the austenite to martensite phase transition, being typically described as stress-induced martensite (SIM). Owing to the high level of exhibited loading-rate sensitivity (Chen *et al.*, 2001 ▸; Nemat-Nasser *et al.*, 2005*a* ▸,*b* ▸; Adharapurapu *et al.*, 2006 ▸), ultrafast diffraction has been used to verify the phase change occurrence during Kolsky bar tensile loading.

**Figure 9:**
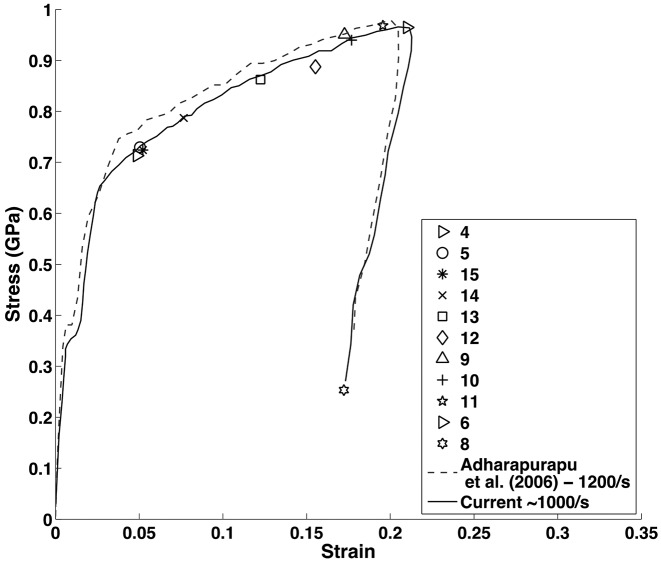
Representative stress–strain curve produced from the equiatomic NiTi super-elastic pulled in tension. Tests were performed at 1000 s^−1^ and are demonstrated by the solid black curve, which is compared with the dotted black line, representing previous data performed at ∼1200 s^−1^ (Adharapurapu *et al.*, 2006 ▸). Similar to the aluminium tests, symbols have been overlaid on the plot demarcating stress–strain states at which diffraction patterns have been captured with the ICCD.

Similar to the Al samples, deformation of NiTi has been impinged *via* Kolsky bar loading, resulting in a strain-rate of ∼1000 s^−1^, and the stress–strain response has been recorded and is displayed in Fig. 9 ▸. Only single diffraction patterns could be collected for one loading duration, thus rendering the use of multiple loading sequences necessary in order to build up a representative response from the deformation history. Each of the aforementioned demarcations relates to a specific stress–strain location wherein a diffraction pattern was recorded. In this set of experiments the detector collection time was 3.37 µs, thereby resulting in a collection of 22 X-ray pulses, which is deemed reasonable as the duration of loading is 100–200 µs. The undulator gap was set to 20 mm and the corresponding X-ray energy spectrum is shown in Fig. 1(*b*) ▸.

**Figure 10:**
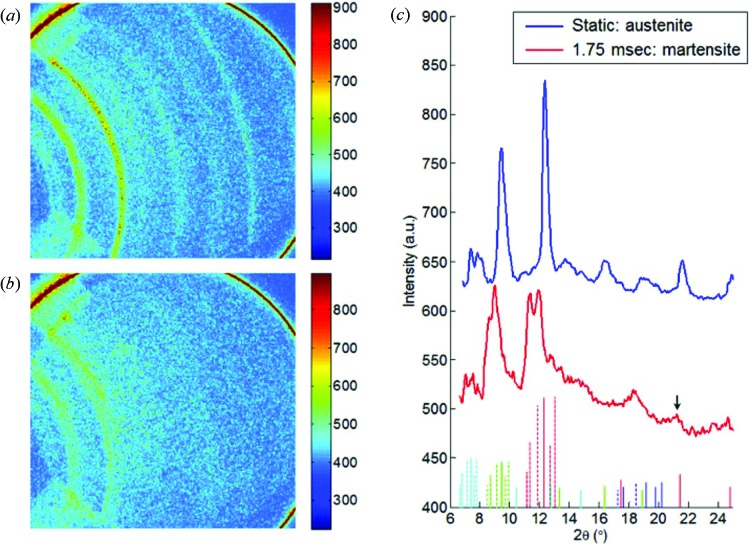
Diffraction patterns from the NiTi sample, collected (*a*) before and (*b*) 1.75 ms after the start of tensile pulling. (*c*) One-dimensional diffraction intensity profiles, obtained by radially averaging the two-dimensional patterns with all available azimuthal angles. Reference peak positions of the austenite (solid lines) and martensite (dashed lines) phases are displayed at the bottom, and the blue, red, green and cyan colors represent peaks that correspond to the second, third, fourth and fifth harmonic energies, respectively. Note that the photons with the first-harmonic energy have been entirely absorbed by the Si filter.

Figs. 10(*a*) and 10(*b*) ▸ show the diffraction patterns from a NiTi sample, collected before loading and 1.75 ms from the start of loading. The difference in the diffraction peak position and intensity clearly indicates a phase transformation of the NiTi sample during the dynamic tensile deformation. Radially averaged one-dimensional diffraction profiles of these two states of NiTi are shown in Fig. 10(*c*) ▸, and they can be well indexed as austenite and martensite phases, respectively. The different colors of the indexing bars represent different X-ray harmonic energies. The solid bars mark the peak positions of the austenite phase, while the dashed bars mark those of the martensite phase. In the diffraction data obtained during the dynamic loading, the small peak around 21° can be observed, as indicated by the arrow, which is attributed to the austenite phase. The presence of this peak indicates the incomplete phase transformation of NiTi at the present situation (Schmahl *et al.*, 2004 ▸). This example of NiTi shown here demonstrates the capability of the ultrafast diffraction technique in probing the transient phenomena in hard crystalline materials, such as rapid phase transformation.

**Figure 11:**
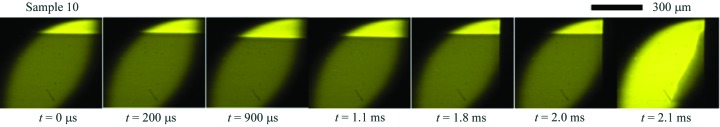
PCI sequence captured with the high-speed camera showing the deformation process of the NiTi material throughout the loading process. Frame times have been chosen to correlate with the stress–strain states at which diffraction patterns displayed in Fig. 12 ▸ were captured. Note that this image sequence consists of snapshots within one loading event.

**Figure 12:**
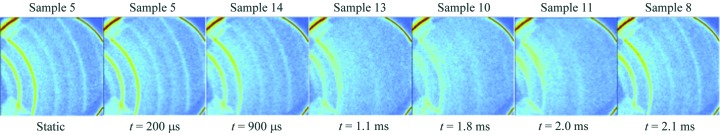
Diffraction pattern sequence captured with the ICCD which shows the evolution of the martensitic transformation at sequential stress states during the dynamic loading process. It is important to note that each pattern is captured from a different sample within the tensile loading history at stress–strain states represented by the appropriate symbols in Fig. 9 ▸ and at delay times shown in Fig. 11 ▸. The pattern taken at *t* = 1.75 ms was captured within the PCI image sequence shown in Fig. 10 ▸.

Figs. 11 ▸ and 12 ▸ are presented to further demonstrate the simultaneous imaging-diffraction nature of these experiments. The former image sequence depicts a set of phase-contrast images derived from a single dynamically loaded NiTi sample being loaded at a ∼1000 s^−1^ strain-rate, while the latter illustrates single diffraction patterns captured from various samples that have been loaded *via* similar conditions. Each image in Fig. 12 ▸ thus presents a diffraction pattern which corresponds to a similar stress-state at which the phase-contrast image presented in Fig. 11 ▸ was collected. While the real-space images could only show limited structural information of the NiTi sample during high-rate loading, the diffraction patterns clearly reveal the phase transformation process. As shown in Fig. 12 ▸, the NiTi transforms from the original austenite phase to the martensite phase (from the third frame to the fourth frame) upon tensile strain, and changed back to the austenite phase after the sample fractured and the strain was released in the end (from the sixth frame to the seventh frame). As captured by the current data set, NiTi clearly undergoes the SIM transformation during dynamic tensile loading in the 1000 s^−1^ strain-rate regime being concretely verified by the slight pattern shift shown in Figs. 10 ▸ and 12 ▸. As shown, the ambient cubic crystal structure reorients to the martensitic phase exhibiting a monoclinic structure during loading and then, upon failure, returns to the initial cubic structure.

Furthermore, the NiTi example shown here is intended to demonstrate that the ultrafast diffraction can well complement the established PCI technique, and help provide additional insight into the material deformation process. Also, it underscores the need of proper analysis tools in understanding the complex white-beam diffraction data. Although the present work required multiple experimental runs to build up a representative diffraction pattern evolution, it can be clearly seen that it is possible to gather fine temporal resolution pattern evolution during the high-rate loading sequence.

## 4. Conclusion   

In order to better understand the deformation process of materials loaded in a dynamic environment, both equiatomic NiTi and aluminium samples have been pulled in tension with a miniature Kolsky bar, while simultaneously performing X-ray PCI and diffraction. Using synchrotron radiation at beamline 32-ID-B of the Advanced Photon Source at Argonne National Laboratory, high-temporal-resolution X-ray diffraction patterns can be generated, which thus allow for legitimate material analysis of dynamically loaded samples. Evolution of sample texture, elastic crystal straining and material phase can all be analyzed using in-house software (*WBXRD_GUI*), with the latter two being immediately confirmed in this work. Furthermore, with the described experiments and data analysis, it has been shown that white-beam diffraction is sufficient to perform the aforementioned investigation. While the majority of this work displays a diffraction pattern temporal resolution of 3.37 µs, it has also been shown that a resolution of 100 ps is possible using the standard 24-bunch mode offered at APS.

## Figures and Tables

**Table 1:** Experimental parameters for each of the chosen conditions. Note the high level of temporal resolution

	Aluminium		
	Short exposure	Long exposure	NiTi
Undulator gap (mm)	30	30	20
Detector angle (°)	25	25	15
Shutter time (µs)	0.153	3.37	3.37
Temporal resolution (ns)	100 ps	3370	3370
Number of X-ray pulses	1	22	22
